# Manual acupuncture as prophylaxis for migraine without aura: study protocol for a multi-center, randomized, single-blinded trial

**DOI:** 10.1186/s13063-022-06510-7

**Published:** 2022-07-19

**Authors:** Jiao Chen, Siyuan Zhou, Mingsheng Sun, Yanan Wang, Xiaoqin Chen, Taipin Guo, Lu Liu, Jing Luo, Xixiu Ni, Xuguang Yang, Dehua Li, Shan Gao, Zhenxi He, Dingjun Cai, Ling Zhao

**Affiliations:** 1 Acupuncture and Tuina School, Chengdu University of Traditional Chinese Medicine, No.37 Shier Qiao Road, Chengdu, 610075 Sichuan China; 2 Clinical Research Center for Acupuncture and Moxibustion in Sichuan Province, No.37 Shier Qiao Road, Chengdu, 610075 Sichuan China; 3 Chengdu Pidu District Hospital of Traditional Chinese Medicine, No. 342, South Street, Pidu District, Chengdu, 610075 Sichuan China; 4 Acupuncture and Massage-Rehabilitation School, Yunnan University of Chinese Medicine, No.1076 Yuhua Road, Kunming, 650500 Yunnan China; 5 Hospital (T. C. M) Affiliated to Southwest Medical University, No.182 Chunhui Road, Longmatan District, Luzhou City, 610075 Sichuan China; 6 Third Affiliated Hospital of Henan University of Traditional Chinese Medicine, No.63, Dongming Road, Zhengzhou, 450000 Henan China; 7 Affiliated Hospital of Chengdu University of Traditional Chinese Medicine, No.39 Shier Qiao Road, Chengdu, 610075 Sichuan China

**Keywords:** Migraine without aura, Clinical trial protocol, Randomized controlled trial

## Abstract

**Background:**

Migraine is a highly prevalent neurological disorder. It is the third most prevalent disorder and the seventh highest cause of disability worldwide. Acupuncture may be a viable prophylactic treatment option for frequent or uncontrolled migraine. Clinical studies comparing acupuncture and placebo acupuncture have not reached a consistent conclusion in confirming whether acupuncture is effective in migraine prophylaxis. The effect of acupuncture mainly depends on acupoints and needles operation. We found that the design of the placebo acupuncture in previous studies included shallow needling at sham acupoints, non-penetrating needling at sham acupoints, and needling at inactive acupuncture points to achieve the inert effect of control group, but the non-penetrating needling at true acupoints was ignored. This randomized controlled trial aims to use true acupoints for non-penetrating acupuncture as control to evaluate the efficacy of manual acupuncture for the prophylaxis of migraine without aura (MWoA).

**Methods/design:**

This is a single-blinded, randomized, controlled, prospective, multi-center trial with two parallel treatment groups. A total of 198 eligible patients with MWoA will be randomly divided into two groups (1:1 allocation ratio). The intervention group will receive manual acupuncture and the control group will receive placebo acupuncture (non-penetrating). Patients will receive three acupuncture treatment sessions per week for 4 consecutive weeks. All patients will then receive a 12-week follow-up.

**Discussion:**

In this study, we are evaluating the efficacy and safety of manual acupuncture in the prophylaxis of MWoA. The placebo control is using non-penetrating needling verum acupoints. It is essential to determine an appropriate control method to ensure the methodological quality of a randomized controlled trial.

**Trial registration:**

The trial has been registered in the Chinese Clinical Trial Registry (approval no. ChiCTR2000032308) in April 2020.

**Supplementary Information:**

The online version contains supplementary material available at 10.1186/s13063-022-06510-7.

## Background

Migraine is a highly prevalent brain disorder that has serious effects on socioeconomic factors and health [1]. Migraine is the third most prevalent disorder and the seventh highest cause of disability worldwide. The Global Burden of Disease Study reported that migraine is the second highest cause of years lost because of disability and interferes considerably with occupational, educational, family, and social responsibilities [2]. In Europe, one third of the population presents with at least one brain disorder, including migraine, resulting in an overall cost of 798 billion euros per year [3]. In East Asian adults, the 1-year prevalence of migraine among adults is 6.0 to 14.3%. Peak prevalence is 11 to 20% for women and 3 to 8% for men (30- to 49-year-olds). The peak prevalence in older people is 1.2% among 60- to 69-year-olds in China [4].

Acupuncture is the main non-pharmacological therapy of traditional Chinese medicine (TCM) and is superior in long-term effects and few side effect in migraine prophylaxis [5, 6]. On the basis of evidence from systematic reviews and meta-analyses, acupuncture could be a viable prophylactic treatment option for frequent or uncontrolled migraine, or for migraineurs experiencing drug side effects [7, 8].

But in terms of demonstrating that migraineur benefits from acupuncture treatment beyond placebo effects, findings from placebo-controlled trials were inconsistent [9–11]. Some reports indicated that the therapeutic effect of verum acupuncture is not superior to that of sham acupuncture [12–14]. A Cochrane review indicated that acupuncture was effective in reducing migraine attacks, despite the efficacy of acupuncture was over sham, but this effect was still small [7].

We found that the design of the placebo acupuncture in previous studies included the following: (i) superficial needling of the sham acupoints (needing “outside” of acupuncture points) [11]; (ii) placebo needles (use of devices which mimic acupuncture but where skin penetration does not occur) of the sham acupoints [15]; and (iii) inactive acupuncture points (needling of common acupuncture points which, however, were considered ineffective for the condition treated) [16, 17]. However, the non-penetrating needling at true acupoints was ignored in migraine study [16]. There is evidence that sham acupuncture on non-points cannot be considered as physiologically inert [18]. Meanwhile, for chronic pain, non-penetrating placebo control was more appropriate than penetrating placebo control to generate inert and ineffective effect [19].

We designed a single-blind, multi-center randomized clinical trial among patients with migraine who were naïve to acupuncture, using non-penetrating needling verum acupoints as placebo control to evaluate the efficacy of manual acupuncture in the prophylaxis of MWoA.

## Methods and analysis

### Study design

**Fig. 1:**
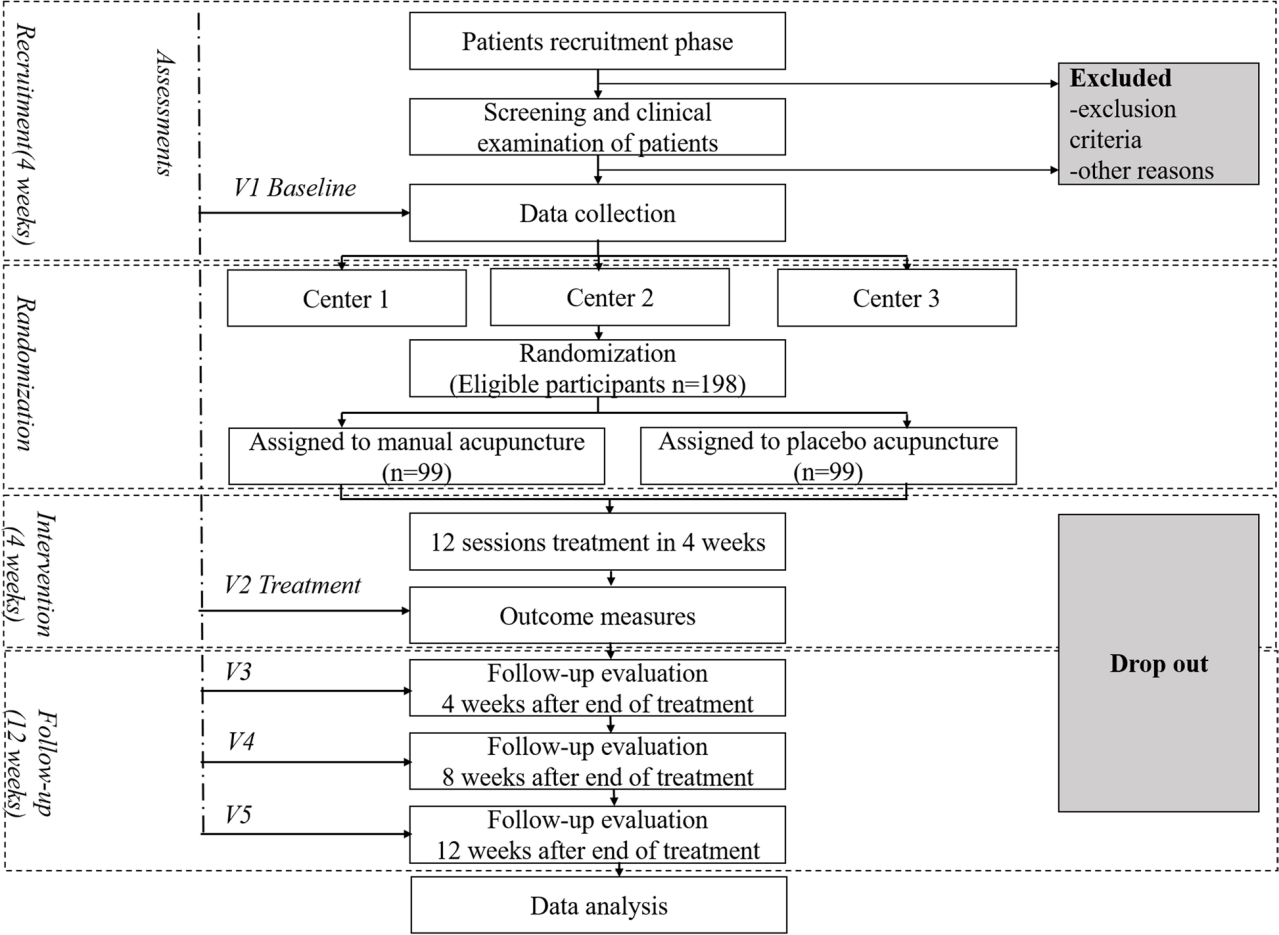
The flow diagram. Patients with a diagnosis of migraine without aura will be recruited at three centers (enrollment areas of Chengdu, Luzhou and Henan) taking part in the study. All participants should endure a baseline period of 4 weeks and inappropriate participants will be excluded. A total of 198 patients will be randomized to two groups: manual acupuncture and placebo acupuncture. Each patient will receive four weeks of treatment and 12 weeks of follow-up

The proposed study is a single-blinded, randomized, controlled, prospective, multi-center trial comprising two parallel treatment groups. A total of 198 patients with MWoA will be assigned to one of two groups (manual acupuncture or placebo acupuncture) using central randomization (a ratio of 1:1). The trial will compare the treatment efficacy and safety of a manual acupuncture group and a placebo acupuncture group. The study duration will be 20 weeks for all participants. This period includes a 4-week baseline period, a 4-week intervention period, and a 12-week follow-up period. The flow diagram of the study procedure is shown in Fig. 1.

**Fig. 2:**
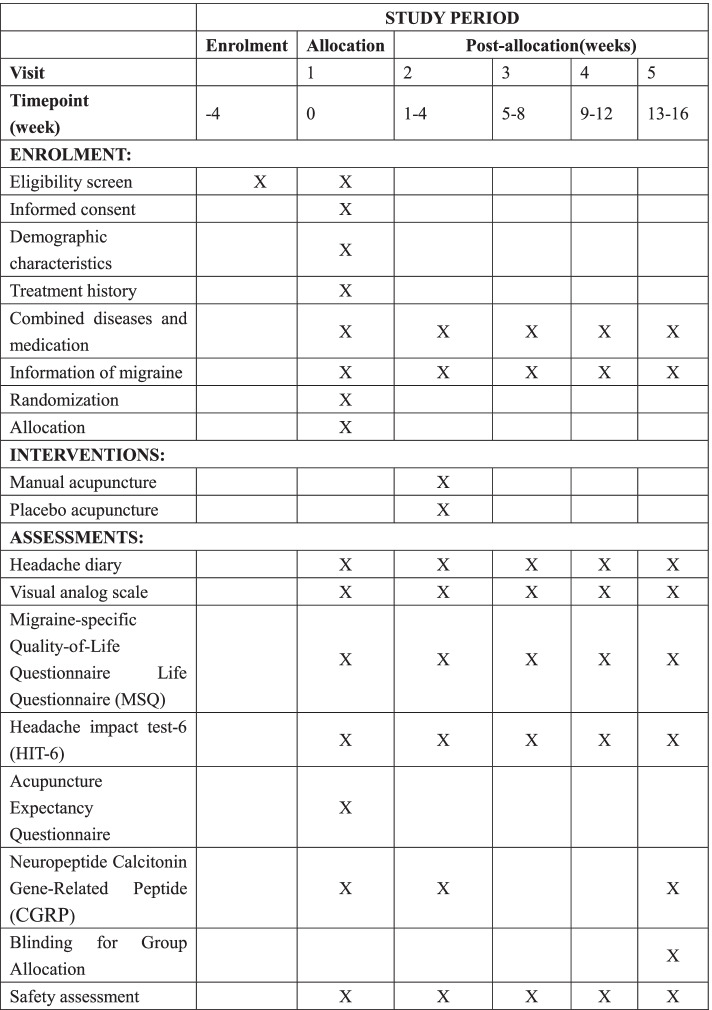
Timetable of enrolment, interventions, and assessments

This study protocol is compliant with the principles of the Consolidated Standards of Reporting Trials (CONSORT) [20] (http://www.consort-state-ment.org/home/) guidelines and the Standards for Reporting Interventions in Clinical Trials of Acupuncture (STRICTA) as well as with the Standard Protocol Items: Recommendations for Intervention Trials (SPIRIT). The SPIRIT checklist is presented in Additional file 1. Figure 2 for the recommended SPIRIT figure (Fig. 2).

The study protocol was approved by the ethics committee of the hospital of Chengdu University of Traditional Chinese Medicine (Chengdu, China) in March 2020 and is in accordance with the Declaration of Helsinki. The permission number of the trial protocol is 2020KL-003. The trial has been registered in the Chinese Clinical Trial Registry (Approval No. ChiCTR2000032308). Written informed consent will be obtained from each patient before they begin the trial.

### Randomization and concealment

Central randomization will be conducted using the Clinical Information Management System (CIMS) (website: https://apps.cims-medtech.com/C028/). Allocation to treatment groups uses a stratified block dynamic randomization method with permuted block, which is under the automatic control of a central computer system. To guarantee allocation concealment, randomization will be carried out by an independent researcher. A website message will be used to send randomization information (including the center number, the patient’s name in *pinyin* initials, and the screening number) to the CIMS center. An independent assessor will interview the participants and carry out the screening. Random numbers and group assignment will be confirmed immediately by email to the independent assessor. This procedure will ensure that randomization concealment is adequate and not influenced by the acupuncturists or participants.

### Blinding and informed consent

Patients will be informed that they have a 50% chance of being allocated to one of the two treatments: manual acupuncture or placebo acupuncture. The placebo acupuncture will generate the same stimulation as the manual acupuncture. Therefore, patients will be blinded to their treatment allocation.

We will use non-penetrating needles as the control, and as far as possible, the procedures in the manual acupuncture and placebo acupuncture groups will be the same. Patients from different groups will be prevented from communicating with each other. The success of blinding will be tested at the end of the follow-up (week 20). Furthermore, outcome assessors and personnel involved in data collection and statistics will be blinded to the treatment allocation throughout the trial. Acupuncturists cannot not be blinded to the treatment allocation because of the nature of the interventions, but they will be asked not to communicate with participants or outcome assessors about treatment procedures and responses.

All patients will participate in a standardized interview and will receive details of the study (e.g., the possible risks associated with the study) and asked to complete a headache diary. Participants will be advised on their right to withdraw from the trial at any time without specifying reasons, and will provide written informed consent for their voluntary participation before enrollment.

### Patient recruitment

Trial participants with migraine are being recruited by clinicians from outpatient clinics at four clinical centers: the hospital of Chengdu University of TCM, Southwest Medical University Affiliated Hospital of TCM, Chengdu Pidu District Hospital of TCM, and the third affiliated Hospital of Henan University of TCM. Detailed information about the trial is being posted at the outpatient clinics, on the WeChat platform (WeChat, version 6.7.3, Tencent, Shenzhen, China), and on the official hospital websites to ensure greater dissemination.

### Inclusion criteria

To be eligible, patients should meet the MWoA diagnostic criteria in the International Classification of Headache Disorders [21] and the guidelines of the International Headache Society for controlled trials of preventive treatment of migraine attacks in adults with episodic migraine [22]. They must (i) be aged between 18 and 55 years, with initial onset of migraines before the age of 50 years; (ii) have had two to eight migraine attacks, but less than 15 days of attacks per month, during the last 3 months and the baseline period; (iii) have experienced migraine attacks for more than 1 year; (iv) have completed the headache diary and provided baseline values in the diary; (v) have a visual analog scale (VAS) pain score of 3 to 7 during the baseline period; (vi) have not received any acupuncture treatment in the preceding 3 months; and (vii) personally provided written informed consent.

### Exclusion criteria

Individuals who meet any of the following criteria will be excluded from the trial: (i) tension-type headache, cluster headache, or other primary headaches; secondary headache disorders; neuralgia of the face or head; (ii) relatively severe systemic diseases (cardiovascular disease, acute infectious disease, hematopathy, endocrinopathy, allergy or hyperthyroidism); (iii) severe mental illness, such as severe anxiety and depression; (iv) pregnancy, lactation, or insufficient contraception; (v) involvement in other clinical trials; and (vi) inability to read and understand the evaluation scales.

### Dropout criteria

Any participant will be dropped from the study if he or she (a) has a belated discovery of any ineligible criteria; (b) withdraws consent, wants to cease, or refuses to undergo the *manual acupuncture* or placebo acupuncture procedures; (c) is lost to follow-up; (d) shows serious adverse events and fails to maintain the protocol; (e) takes concomitant use of the prohibited drugs or therapies; (f ) violates the protocol seriously; or (g) is considered inappropriate to keep in the trial by investigators’ clinical experience. The close contact due to the two or three intervention sessions per week is expected to promote patient adherence and minimize the dropout rate. In addition, we will personally remind the participants by telephone before each follow-up appointment.

### Interventions

Treatment strategies were determined according to TCM theory and the results of our previous research on acupuncture treatment for migraine [23]. There will be two groups in this trial: a manual acupuncture group and a placebo acupuncture group. The Park sham device (PSD) will be used to administer both manual acupuncture and placebo acupuncture. The PSD comprises a ring-base unit and a special oversized tube (a Park tube). The ring-base of the device is kept in place on the participant’s skin using double-sided sticky tape. The internal circumference of the ring-base fits tightly around the Park tube [24]. This form of placebo acupuncture may be considered an advance on all previously used methods. Patients in both groups will receive 12 sessions of acupuncture (30 minutes per session) over a 4-week period (three acupuncture sessions per week).

Acupuncture will be performed by experienced acupuncturists with more than 5 years clinical experience in acupuncture treatment. All acupuncturists will receive special training regarding the trial purpose and standard procedure, treatment strategies, and quality control. To facilitate replication of the intervention, the location and manipulation of the acupoints that will be used are shown in Table 1.

**Table 1:** Acupuncture treatment

Point selection	Pain localization	Acupuncture points	Depth [cun]
Standard acupoint		Baihui (GV20)	0.5–0.8
		Shuaigu (GB8)	0.5–0.8
		Fengchi (GB20)	0.8–1.2
Shaoyang meridian	Side head, temporal	Waiguan (TE5)	0.5–1
		Yanglingquan (GB34)	1–1.5
Yangming meridian	Front head, forehead, brow edge	Hegu (LI4)	0.5–1
		Neiting (ST44)	0.5–0.8
Taiyang meridian	Back head, occipital	Houxi (SI3)	0.5–1
		Kunlun (BL60)	0.5–0.8
Jueyin meridian	Top of the head	Neiguan (PC6)	0.5–1
		Taichong (LR3)	0.5–1

#### Manual acupuncture group

The acupoint prescriptions used will be personalized for each participant at the discretion of the acupuncturist. Each prescription will comprise a standardized combination of points plus additional points chosen according to meridian indications and the patient’s symptoms. Syndrome differentiation according to the meridians is an important part of TCM theory and will be used to select acupoints based on the evolution of the patient’s symptoms.

Several standard points will be used for all patients: GV20 (Baihui), bilateral GB8 (Shuaigu), and bilateralGB20 (Fengchi). The following additional bilateral points may be chosen according to meridian syndrome differentiation: for Shaoyang headache (TE-GB): bilateral TE5 (Waiguan) and bilateral GB34 (Yanglingquan); for Yangming headache (LI-ST): bilateral LI4 (Hegu) and bilateral ST44 (Neiting); for Taiyang headache (SI-BL): bilateral SI3 (Houxi) and bilateral BL60 (Kunlun); and for Jueyin headache (PC-LR): bilateral PC6 (Neiguan) and bilateral LR3 (Taichong).

Acupoint localization, selection, and insertion depth will follow TCM theory and practice. Patients will be asked to lie on their backs in a comfortable position and with the chosen acupoints accessible. After disinfection with a 75% alcohol solution, sterile disposable acupuncture needles will be used (length, 25 to 40 mm; diameter, 0.25 to 0.3 mm; manufacturer: Suzhou Huatuo Medical Equipment Co., Ltd, China) in conjunction with the PSD. After exposing the tip of the needle, a suitable needle insertion method will be applied and the ring-base of the device attached to the skin. Acupuncture (rotation or lifting) will be performed every 15 min to evoke a Deqi sensation; each needle will be manipulated manually for 10 s and retained for 30 min.

#### Placebo acupuncture group

This study has been designed to investigate the effect and safety of manual acupuncture compared with a placebo acupuncture control. Therefore, we selected non-penetrating acupuncture using sham needling with the PSD for comparison. Instead of penetrating the skin, the tip of the PSD sham needle is blunt and is retracted into the shaft when pressed against the skin. The sham needles measure 0.25 mm × 30 mm and 0.25 mm × 40 mm. The sham needles will be precisely placed using the PSD at true acupoints. The standard combination of points plus additional individual points identified by meridian indications and patient symptoms will be the same as for the manual acupuncture group.

To ensure patients blinding, the acupuncture procedure will be the same in both the manual and placebo acupuncture groups. During the 30-min session, each needle will be manipulated manually for 10 s. This will be repeated three times, with a 15-min interval. The use of the same acupoints for both the manual and placebo acupuncture groups will help in ensuring patient blinding. At the end of the treatment, the acupuncturist will use a dry cotton ball to press each acupoint so that patients will feel the withdrawal of “real” needles.

Participants in both groups will be asked not to take any prophylactic medications for migraines but will be permitted to use ibuprofen (300 mg capsules with sustained release) as a rescue medication if they have severe migraine pain (VAS score > 8) [17]. The use of ibuprofen will be documented in the headache diary, and we will assessment the impact on the efficacy.

### Outcome measures

Supervised by independent research assistants, patients will complete five headache diaries every 4 weeks on paper or online from baseline to week 16.

#### Primary outcome

The primary outcome is the change in the frequency of migraine attacks during the 4th and 12th weeks after randomization compared with baseline (4 weeks before randomization).

#### Secondary outcomes

The following secondary outcomes will be assessed:

1. The frequency of migraine attacks every 4 weeks, from baseline to endpoints;
2. The number of days with migraine every 4 weeks, from baseline to endpoints;
3. The VAS score every 4 weeks, from baseline to endpoints;
4. The mean duration of migraine attacks every 4 weeks, from baseline to endpoints;
5. The proportion of the intake dose of rescue medication every 4 weeks, from baseline to endpoints;
6. The proportion of responders, defined as the proportion of patients with at least a 50% reduction in the number of migraine attacks in the 4thand 16th weeks;
7. The change in health-related quality of life, which will be measured using the Migraine-Specific Quality of Life Questionnaire (MSQ) every 4 weeks, from baseline to endpoints;
8. The change in the calcitonin gene-related peptide (CGRP) at baseline, the 4th week, and the 16th week;
9. To test the blinding for group allocation, patients will be asked at the end of the study to guess which type of acupuncture therapy they had received;
10. The level of acupuncture expectation measured by acupuncture expectancy scale at baseline;
11. The number of patients with adverse events and serious adverse events every 4 weeks, from baseline to endpoints;
12. Evaluation of patients’ satisfaction with the treatment at the end of the study.

Blood samples will be collected on the clinical center between 8 and 10 am, after overnight fasting. In addition, blood samples collection should be arranged on the 1st or 2nd day of each time node as far as possible, but avoid menstrual period. All physicians who enroll participants and assessors who collect data must attend training to ensure the use of identical practices at each clinical center. The training classes will comprise theoretical and practical lessons. Physicians must pass a test that assesses whether they have understood the purpose and content of the trial, treatment strategies, and quality control. Additionally, to maintain quality control, quality monitoring will be carried out monthly by inspectors from each research center, and specially trained physicians will check all trial processes. Dropout and withdrawal reasons and details will be documented. Details of the outcome assessment time points are shown in Table 2.

**Table 2:** Timetable of enrolment, interventions, and assessments

	Study period					
	Baseline	Allocation	Treatment	Follow-up phase		
Visit		1	2	3	4	5
Timepoint (week)	− 4	0	1–4	5–8	9–12	13–16
**Enrolment:**						
Eligibility screen	X	X				
Informed consent		X				
Demographic characteristics		X				
Treatment history		X				
Combined diseases and medication		X	X	X	X	X
Information of migraine		X	X	X	X	X
Randomization		X				
Allocation		X				
**Interventions:**						
Manual acupuncture			X			
Placebo acupuncture			X			
**Assessments:**						
Headache diary		X	X	X	X	X
Visual analog scale		X	X	X	X	X
Migraine-specific Quality-of-Life Questionnaire Life Questionnaire (MSQ)		X	X	X	X	X
Headache impact test-6 (HIT-6)		X	X	X	X	X
Acupuncture Expectancy Questionnaire		X				
Neuropeptide calcitonin gene-related peptide (CGRP)		X	X			X
Blinding for group allocation						X
Safety assessment		X	X	X	X	X

We will set up data monitoring committees (DMCs). These will protect trial participants by ensuring that they are not unduly or unfairly at risk of harm and ensure that the scientific integrity of the trial is maintained. The DMC is an independent advisory group that is essential to ensure unbiased assessment of accumulating trial data. The DMCs, which generally comprise medical and statistical experts with experience in clinical trials, will be developed in accordance with the World Health Organization Operational Guidelines for the Establishment and Functioning of Data and Safety Monitoring Boards. The DMCs will meet periodically to review reports, produced by a statistical data analysis center, which will summarize important interim data [25].

## Data collection and management

All medical information will be documented and stored separately from personally identifiable data. The data will be recorded on the paper version of case report forms and double entered into electronic data capture system hosted at CIMS center by outcome assessors. The data sheets will be coded, and the group allocation will not be visible to third parties. Only pseudonymized data will be used for analysis and only the principal investigators (PI) and statisticians will be allowed to access the final trial dataset to preserve patient confidentiality. The acupuncturist, neurologist, efficacy evaluator, and statisticians will not have access to these data during patient evaluations. If subjects withdraw from the study, no further data will be collected from them. Data already obtained will be used in the intention-to-treat analysis with the consent of the patient or other-wise completely deleted from the database.

### Sample size estimation and statistical analyses

#### Sample size estimation

We designed this trial to determine whether there is a difference between a manual acupuncture group and a placebo acupuncture group in frequency of migraine attacks. On the basis of our previous study [11], we anticipate that the mean frequency of migraine attacks in the acupuncture group will be 3 and in the placebo acupuncture group will be 2. We predict that the common standard deviation of the two groups will be 2.

With a 2-sided significance level of 5% and power of 90%, 86 patients are required for each group. Given an estimated loss-to-follow-up rate of 15%, we plan to enroll 198 patients in total, 99 patients in each group. The sample size calculation was performed using PASS V.15.0 software (SPSS, Chicago, IL, USA).

#### Statistical analyses

All data in this trial will be analyzed using SAS v9.3 (SAS, Cary, NC, USA) and the CIMS by Chengdu CIMS Medical Technology Co., Ltd. The statistician will be blinded to group allocation until the final unblinding.

For the final outcome analysis, all pairwise comparisons will be performed using a general linear model adjusted for baseline values, age, sex, clinical center, and disease course. The comparison between the manual acupuncture group and placebo acupuncture group is the primary outcome of interest in this study. The general summary of differences will be in accordance with CONSORT guidelines, and will use effect size estimates and associated confidence intervals.

The baseline characteristics and clinical outcomes described are based on the intention-to-treat (ITT) population, which will include participants with at least one treatment and one primary outcome measure. Missing data will be replaced according to the last-observation-carried-forward principle. In addition, multiple imputation will be considered when necessary. Data for the per-protocol (PP) set population will also be analyzed. The results of the ITT and PP analyses will be compared to determine if they are consistent. Repeated measures analysis of variance will be used for numerical variables. The chi-square test will be used to analyze categorical variables. Values of *P*< 0.05 will be considered significant.

#### Patient and public involvement

Neither the patients nor the public was involved in the development of the research questions, selection of outcome measures, study design, or study conduct.

## Discussion

This study protocol describes a multicenter, randomized, patient-and-assessor-blinded, parallel clinical trial, which will investigate the effect and safety of manual acupuncture compared with non-penetrating placebo acupuncture for MWoA. It is essential to determine an appropriate control method to ensure the methodological quality of a randomized controlled trial. However, it is challenging to design an inert placebo control for acupuncture because acupuncture is a physical intervention. Previous studies of migraine [11, 16] have used minimal acupuncture (using fine needles to superficially penetrate the skin) at non-acupoints as a method of placebo control. However, some researchers have advised that the use of sham acupuncture as a control tends to produce more “negative” results than the use of verum acupuncture versus other types of control, such as placebo acupuncture (non-insertion) and non-acupuncture treatment [19, 26]. In addition, sham acupuncture produces a larger effect size than pharmacological placebo compared with non-treatment [27–29]. It has been suggested that sham acupuncture needle insertion produces a true placebo effect and other physiological effects [29].

As it does not penetrate the skin, the PSD produces fewer physiological effects than shamacupuncture [30]. In pervious study, PSD was used as a placebo acupuncture and it had a methodological advantage over the other sham acupuncture methods of the previous studies. In our study, we only use patient-and-assessor-blinded. It is difficult to use genuine double-blinding in acupuncture trials since the acupuncturists must be aware of the participant’s group allocation to deliver the treatment. In both groups, the same acupoints will be used.

This is easier to achieve single blinded, as the procedure using PSD is indistinguishable from the same procedure between manual acupuncture and placebo acupuncture. All outcome assessors and statisticians will be kept unaware of group allocation throughout the whole process. Only the acupuncturists will not be blinded, but they will be required to minimize conversation about the treatment details.

We expect to find the difference in the manual acupuncture group for all outcomes in comparison with the placebo acupuncture group. Thus, we expect different effects for each group for frequency of pain episodes, the primary endpoint, and for the various secondary endpoints, such as intensity of pain, quality of life, and analgesic consumption. An evaluation of adverse events and their severity are important for safety and for patient acceptance of the treatment. Finally, these treatment modalities will be judged by the participating patients with regard to content and time requirements.

## Trial status

We plan to recruit patients soon. The study design process started in June 2020. We anticipate that the trial will be completed by December 2022.

## Supplementary Information

**Additional file 1.** SPIRIT 2013 checklist: recommended items to address in a clinical trial protocol and related documents.

## Data Availability

Data and materials can be obtained from the corresponding author after the trial.

## Abbreviations

MWoA: Migraine without aura
TCM: Traditional Chinese medicine
CONSORT: Consolidated Standards of Reporting Trials
CIMS: Clinical Information Management System
VAS: Visual analog scale
PSD: Park sham device
MSQ: Migraine-Specific Quality of Life Questionnaire
CGRP: Calcitonin gene-related peptide
DMCs: Data monitoring committees
ITT: Intention-to-treat
PP: Per-protocol
